# Supplementary material for: Analog content-addressable memories with memristors
Source: Nat Commun. 2020 Apr 2;11:1638. doi: 10.1038/s41467-020-15254-4 (PMC7118145; doi:10.1038/s41467-020-15254-4)
Supplement: Supplementary file 1 — Supplementary Information [file 41467_2020_15254_MOESM1_ESM.pdf]

Supplemental Information for "Analog content addressable memories with memristors" by Li *et al.*

Analog content addressable memories with memristors

Can Li[1,*], Catherine E. Graves[1,*], Xia Sheng[1], Darrin Miller[2], Martin Foltin[2], Giacomo Pedretti[1], and John Paul Strachan[1,*]

[1]Hewlett Packard Labs, Hewlett Packard Enterprise, Palo Alto, CA 94304, USA
[2]Silicon Design Lab, Hewlett Packard Enterprise, Fort Collins, CO 80528, USA
[*]Emails: can.li@hpe.com; catherine.graves@hpe.com; john-paul.strachan@hpe.com

Contents

1 Supplementary Figures **2**

2 Supplementary Tables **13**

3 Supplementary Notes **14**

4 Supplementary References **22**

1 Supplementary Figures

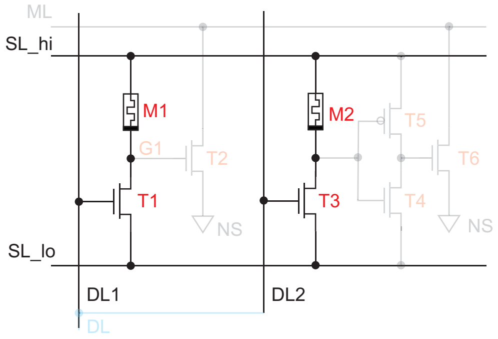

Supplementary Figure 1: The schematic for the programming operation of the memristors in an analog CAM cell

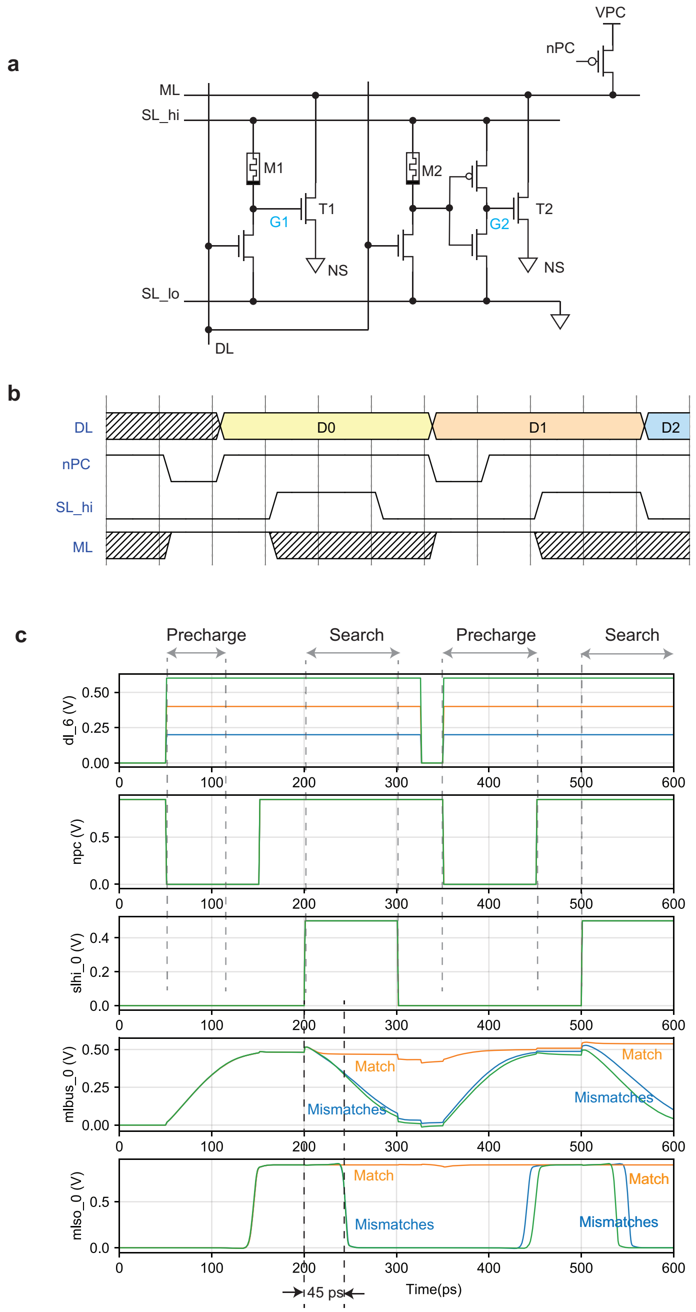

Supplementary Figure 2: Transient circuit simulation for the timing of the search operation. a, A simplified schematic of one analog CAM cell with precharging p-type MOSFET attached to its ML. **b,** The timing diagram for a search operation, where ML precharging is initiated by setting PC high, and the search operation by SL_hi. **c,** Simulated transient plot of the precharging and search operation in a 86×12 analog CAM array for two cycles. The ML is pulled down within 100 ps when the DL voltage mismatches the stored range, and is kept high in the case of a match.

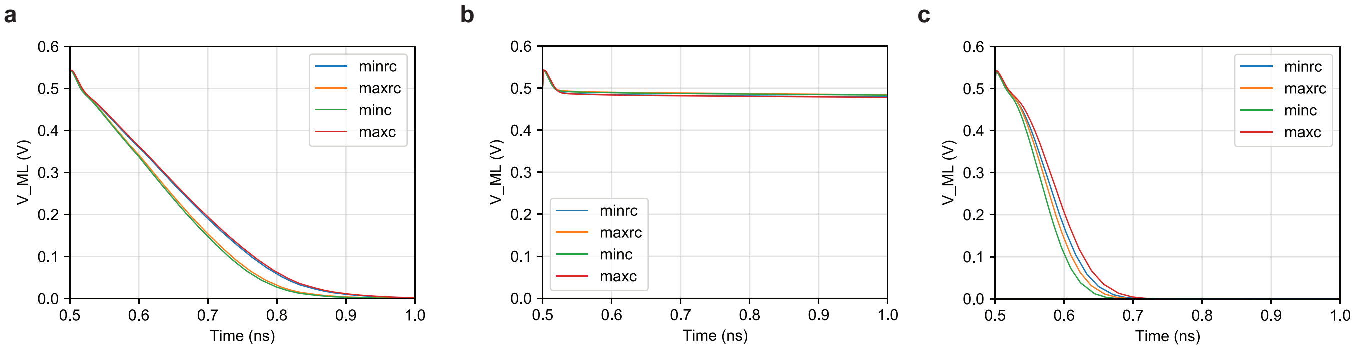

Supplementary Figure 3: Simulation of different process corners. Plots show the ML decay during a search in an 86×12 analog array with DL voltage of **a,** 0.3 V (Mismatch), **b,** 0.4 V (Match), **c,** 0.5 V (Mismatch). The memristor are configured to the same conductance range with that in our single-device simulation in FIGURE 3b.

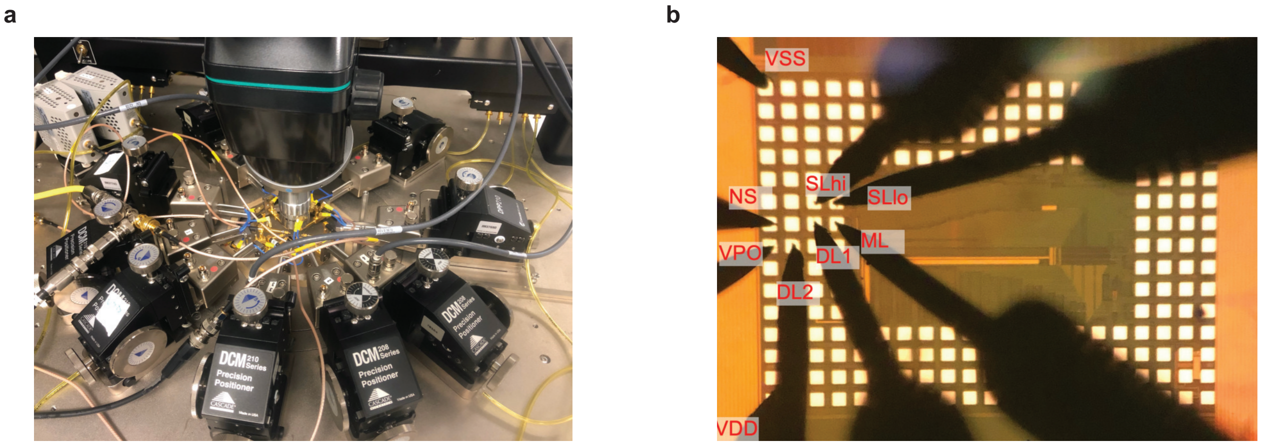

Supplementary Figure 4: The experimental measurement setup. a, The measurement setup with nine probe manipulators on a probe station. **b,** Nine probes landed on the chip under measurement. Each probe is connected to either a source measurement unit (SMU) on Keysight B1500 or a direct-current (DC) voltage supply.

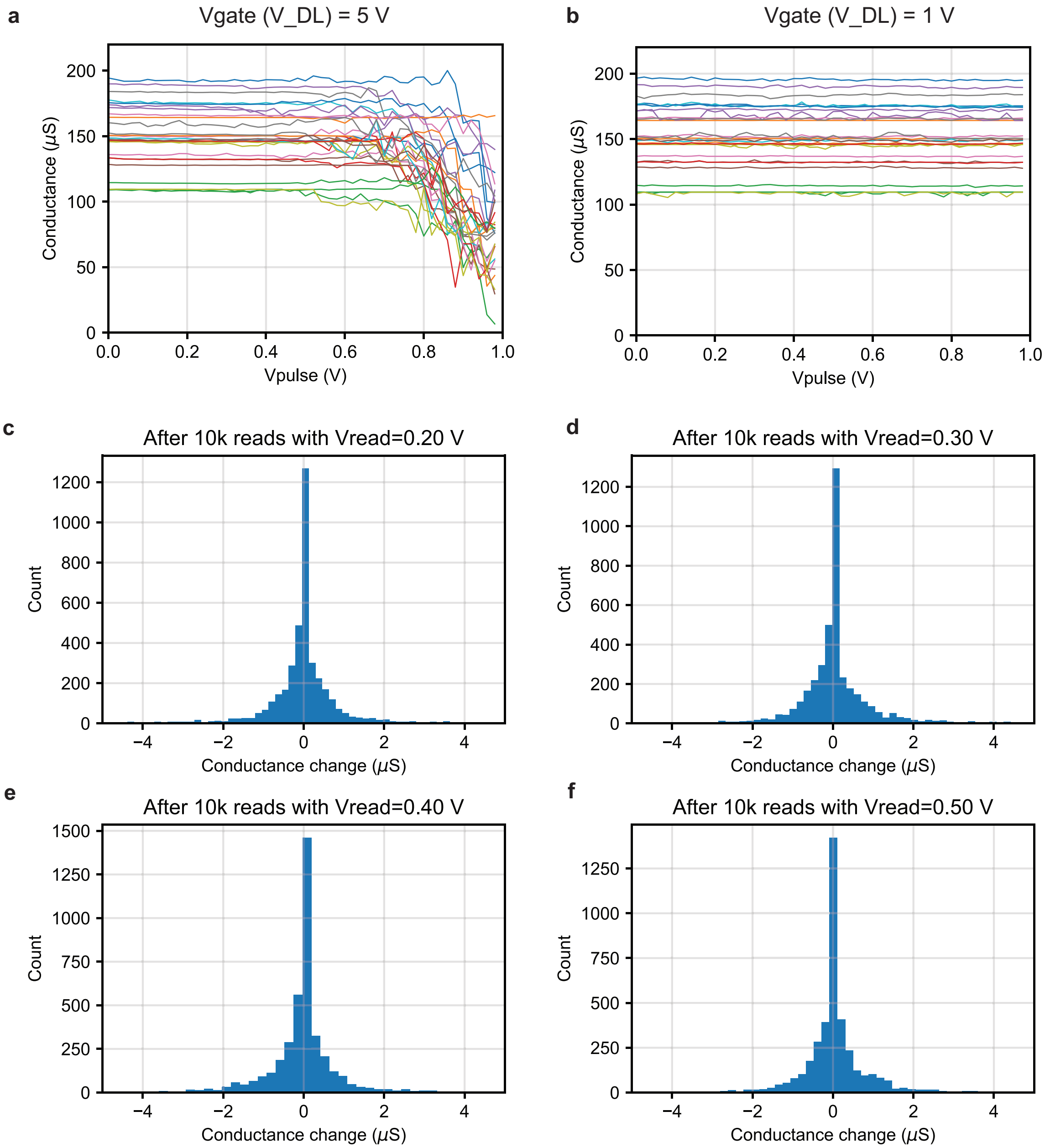

Supplementary Figure 5: Device stability tests with different voltages on SL_hi. a, The memristors are RESET by voltage (>0.5 V) pulses applied to SL_hi with the series transistors fully turned ON. The initial value of the memristors was programmed between 100-200 μS. **b,** On the other hand, the memristor states were not changed by the applied pulses when in the search mode where the V_DL is always smaller than 1 V. **c-f,** The device stability after read operations with different reading voltages. Each panel shows the distribution of the memristor conductance change after 10,000 repeated read operations with the read voltage specified in the title. The conductances do not show noticeable disturb by the read operations, within the noise of the read operation.

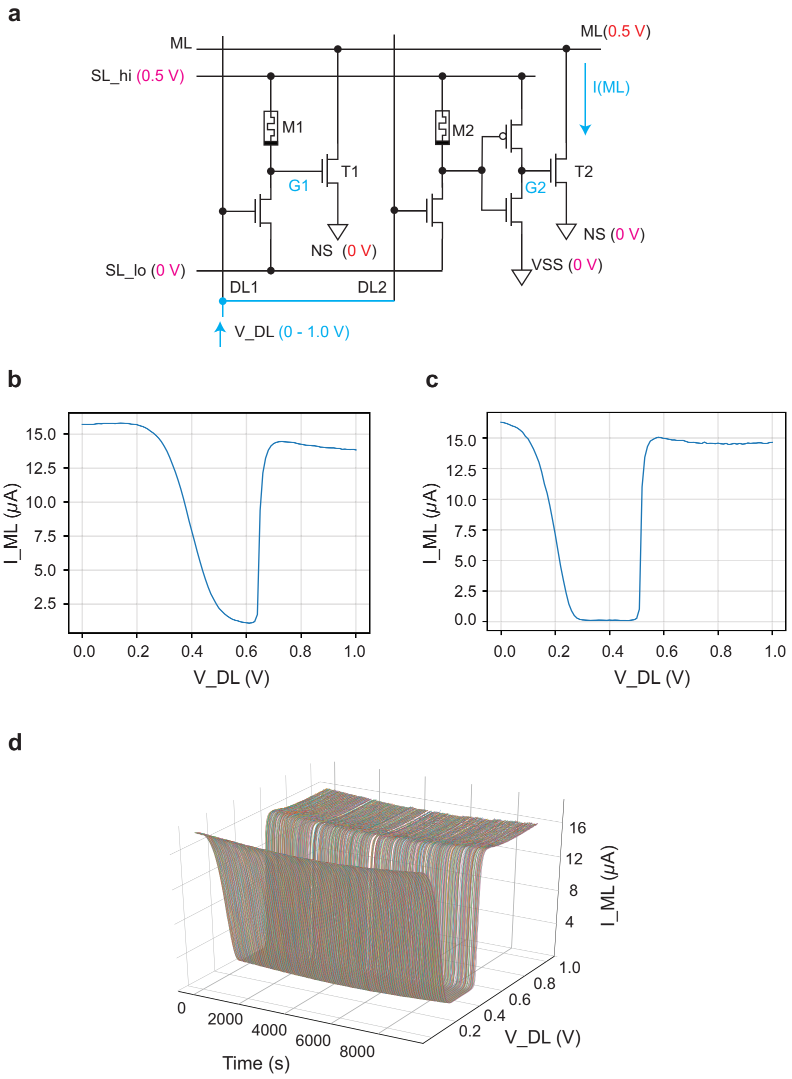

Supplementary Figure 6: Additional information on the experimental measurement. **a,** The schematic shows the measurement setup when measuring the relation between the ML discharge current (I(ML)) and the voltage on DL. The magenta labels mark the DC voltage applied to each node. **b, c,** The ML discharging current with respect to the DL voltage for the two analog CAM cells programmed to search for different ranges. **d,** The retention / reliability test shows that the cell maintains the searching range for more than 8,000 seconds with 1,000 individual measurements. The extracted stored range is shown in FIGURE 4f.

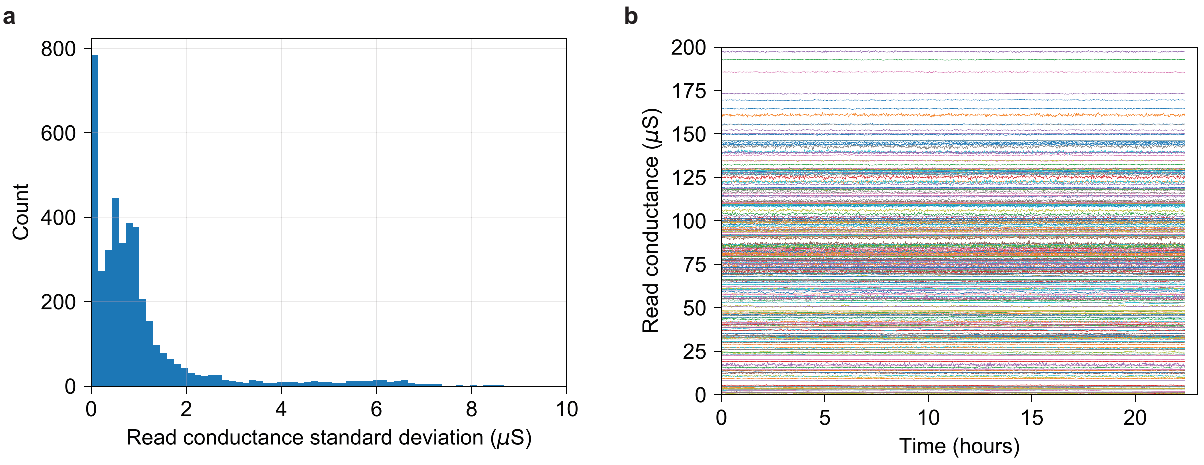

Supplementary Figure 7: Analog conductance state stability and retention. a, Distribution of the memristor device read stability shows that the measured (read) conductance for the majority of the memristor devices has a standard deviation of several μS or smaller. The data was generated from conductance reads with 0.2 V read voltage from all devices in a 64×64 array for 10,000 times. The lateral size of the memristor is 50 nm×50 nm. **b,** Multilevel retention performance of our integrated TaO_x device shows the device conductances did not drift for over 20 hours under room temperature. Each datapoint is averaged from 50 repeated reads.

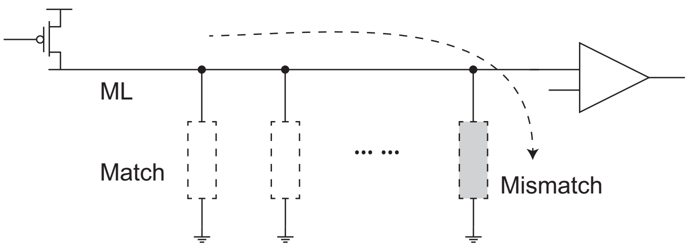

Supplementary Figure 8: The schematic of an analog TCAM word in with only one-bit is mismatch during the search operation.

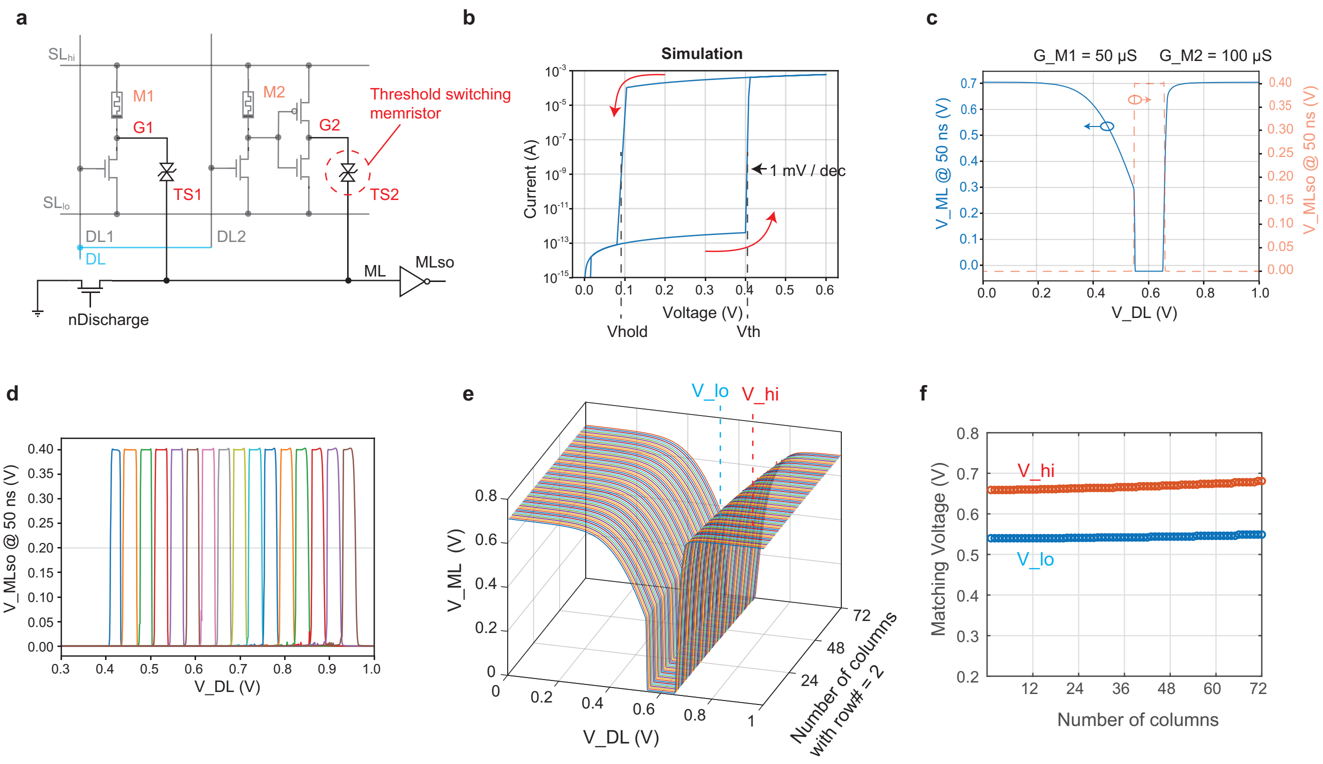

Supplementary Figure 9: An analog CAM circuit using both volatile and non-volatile memristors a, Schematic for our analog CAM circuit, composed of four transistors and four memristors (two non-volatile and two volatile). The volatile threshold switching memristors pull up the ML in the case of a mismatch, which replaces the pull-down transistors in the 6T2M circuit. **b,** The volatile memristor is a threshold switching device with a very sharp transition between states (*e.g.*1 mV/dec[3]), therefore reducing column interference issues exposed in earlier simulations. **c,** The match line stays low only when the input pattern (V_{DL}) matches the stored range. The dashed line shows the signal after the match line sense amplifier output (V_{MLSO}), which inverts and converts the analog signal to a binary 'match' (high) or 'mismatch' (low) signal. **d,** Due to the much smaller $\partial G_{pu}/\partial V_G$, the cell promises the capability to store more accurate ranges and accordingly more bits of discrete levels (showing 16 levels). **e, f** The search operation with a simulated array of different word width. The programmed memristor configuration and the expected searching range is the same as that in FIGURE 5, but the searching range is altered less than 10 mV indicating the capability to store 5-bits of information, which is close to the precision limit of most non-volatile memristor devices.

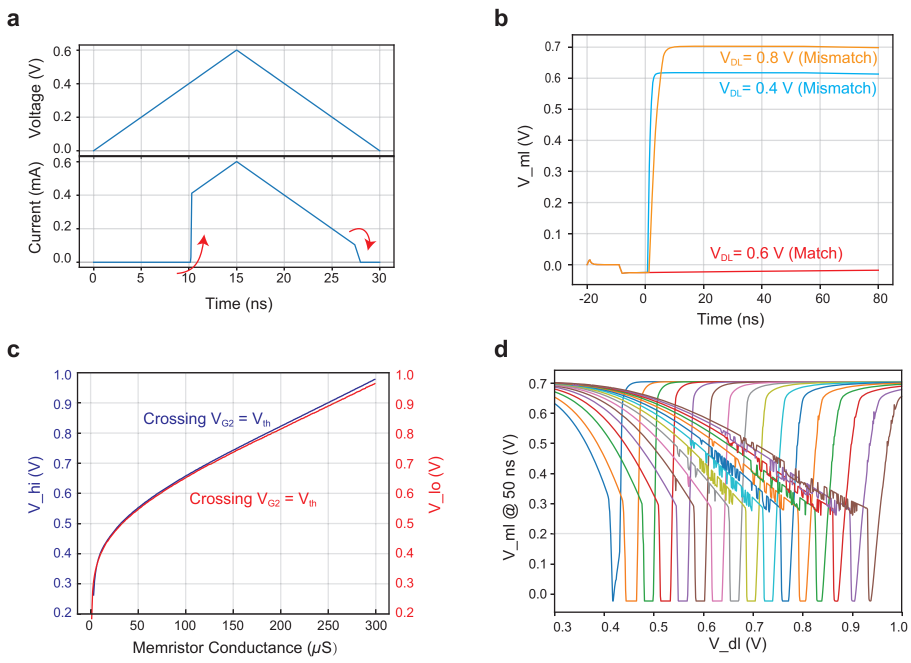

Supplementary Figure 10: Additional information on the analog CAM with threshold switching memristor. a, The transient response of the simulated threshold switching device. The applied voltage is swept from 0 V to 0.6 V. The current flowing through the device abruptly increases when the voltage reached the threshold voltage (0.4 V in this case), and decreases abruptly after the voltage drops below the hold voltage (0.1 V in this case). **b,** The transient voltage response on the match line (ML) during the search operation, for the cases that the stored range matches (in red) and mismatches (in blue and yellow) the input (V_{DL}) respectively. **c,** The relation between the searching ranges and the corresponding memristor conductance. **d,** The match line readout voltage at the time of 50 ns after the search operation for differently configured searching ranges. The corresponding output after a sense amplifier is plotted in FIGURE 9d. The plots show the device can be used to store and search at least 16 discrete levles.

a

21 × 16

0000000110000001
000000011000001X
00000001100001XX
0000000110001XXX
000000011001XXXX
00000001101XXXXX
0000000111XXXXXX
0000001XXXXXXXXX
000001XXXXXXXXXX
00001XXXXXXXXXXX
0001XXXXXXXXXXXX
001XXXXXXXXXXXXX
01XXXXXXXXXXXXXX
1XXXXXXXXXXXXXXX
10XXXXXXXXXXXXXX
110XXXXXXXXXXXXX
111000XXXXXXXXXX
11100100XXXXXXXX
11100100100000XX
111001001000010X
1110010010000110

b

9 × 6

0	0	0	6	0	{1–7}
0	0	0	6	{1–7}	X
0	0	0	7	X	X
0	0	{1–7}	X	X	X
0	{1–7}	X	X	X	X
1	{0–5}	X	X	X	X
1	6	{0–1}	X	X	X
1	6	2	{0–3}	X	X
1	6	2	4	0	{0–6}

c

6 × 4

0	1	8	{1–f}
0	1	9	X
0	{2–f}	X	X
{1–d}	X	X	X
e	{0–4}	X	X
e	5	0	{0–6}

d

3 × 2

01	{81~ff}
{02–e4}	X
e5	{00–06}

Supplementary Figure 11: CAM tables for searching a range between 385 and 58630, with (a) TCAM, (b) 3-bit analog CAM, (c) 4-bit analog CAM and (d) 8-bit analog CAM.

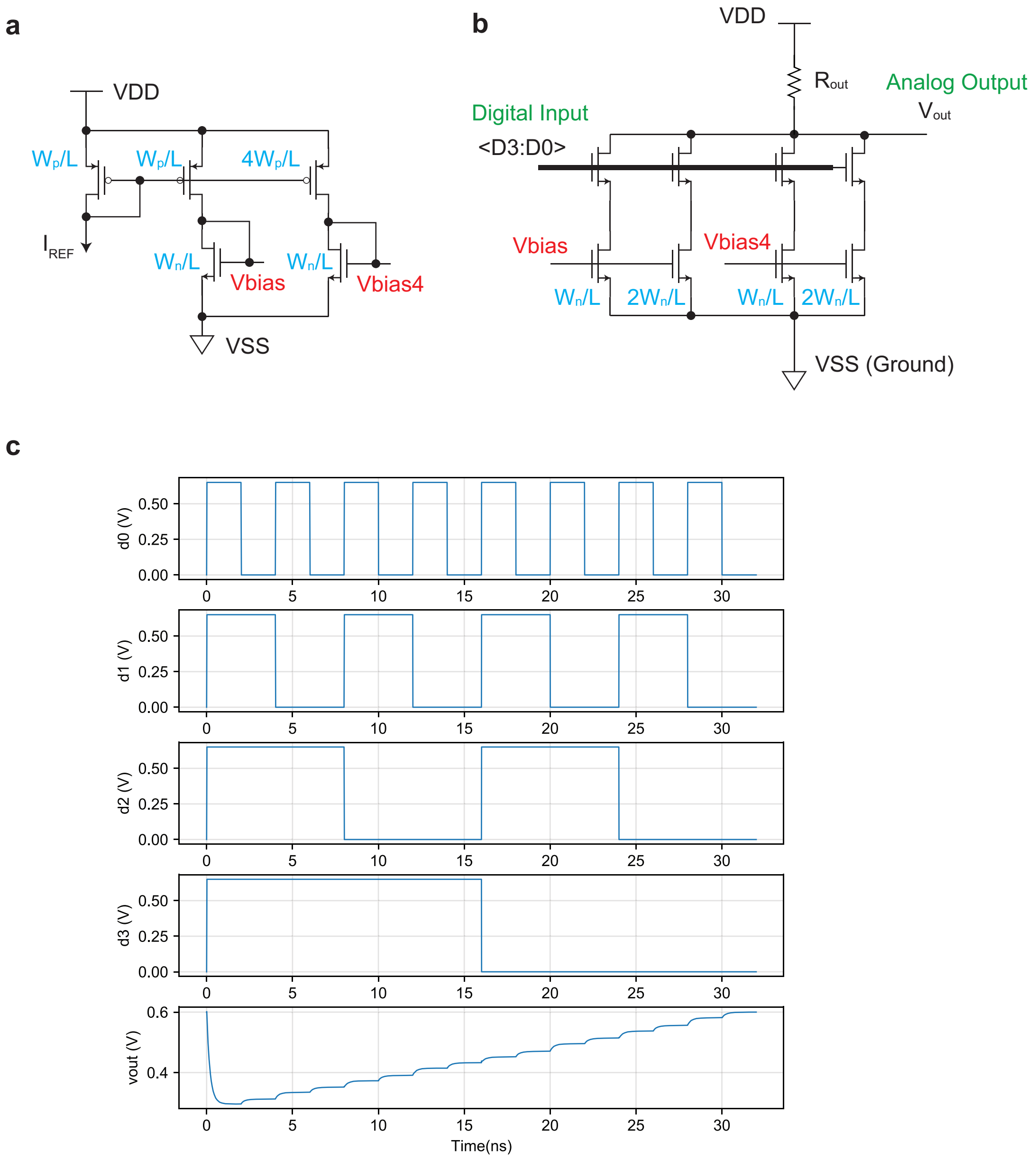

Supplementary Figure 12: A DAC design example for analog CAM. The circuit schematic for the modified current-sterring DAC design with **a,** shared input current mirrors for I_{REF} and $I_{\mathrm{REF}} \times 4$ and **b,** the output current mirrors that converts the digital inputs to the analog output signal. **c,** The simulated DAC operation with different digital inputs.

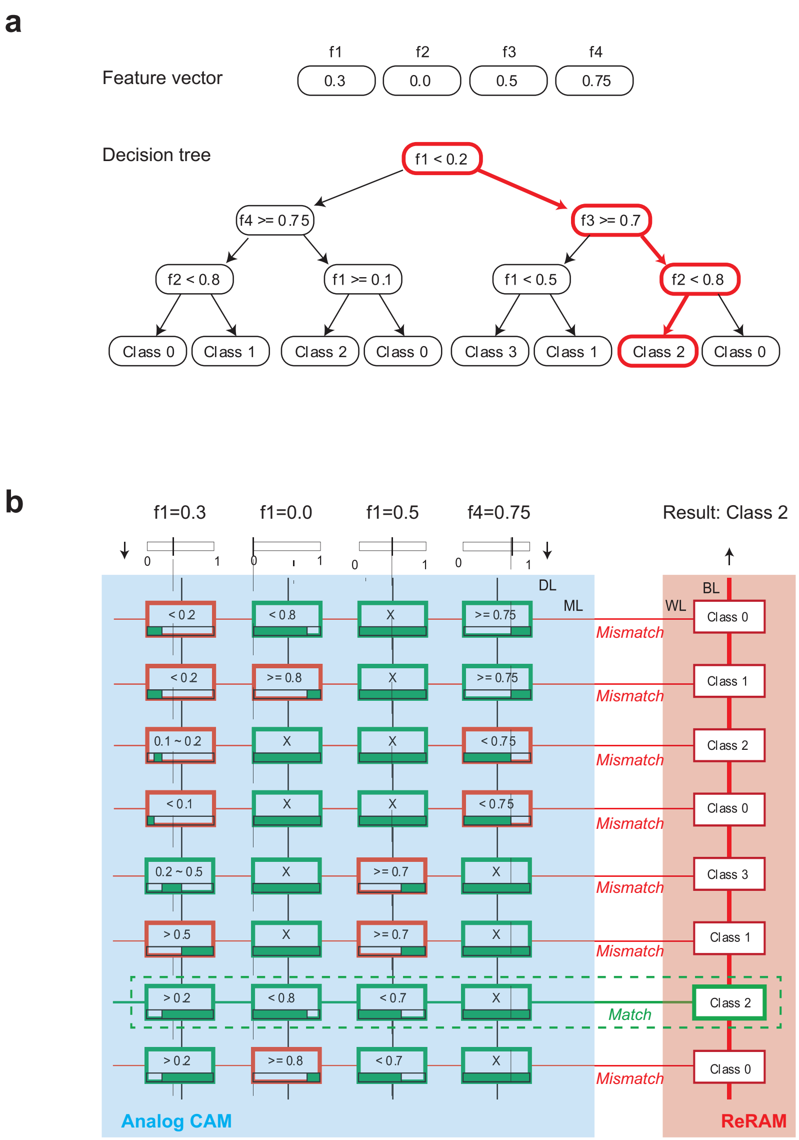

Supplementary Figure 13: Decision Tree in an analog CAM and a memristor RAM for fast tree traversal. (a) The tree structure of a sample decision tree directly mapped to a (b) memristor analog content addressable memory (CAM) in conjunction with a memristor random access memory (RAM).

2 Supplementary Tables

Operation	SL_hi	SL_lo	DL1	DL2
Set M1	Vset	0	Vg,set	0
Reset M1	0	Vreset	V_{DD}	0
Set M2	Vset	0	0	Vg,set
Reset M2	0	Vreset	0	V_{DD}
Read M1	Vread	0	V_{DD}	0
Read M2	Vread	0	0	V_{DD}

Supplementary Table 1: Write operation of the analog CAM cell

Supply	Energy/search	Energy/search/cell
ML precharging	102.9 fJ	0.10 fJ
SLhi driver	298.5 fJ	0.29 fJ
Others	86.4 fJ	0.08 fJ
DAC	52.1 fJ	0.05 fJ
Total	539.9 fJ	0.52 fJ

Supplementary Table 2: Energy break down during searches in a 86 $\times$ 12 array

3 Supplementary Notes

Supplementary Note 1. Programming of memristor devices in an analog CAM array

The memristors in the analog CAM need to be properly programmed before the search operation, and the circuit search operation has been introduced in the main text. SUPPLEMENTARY FIGURE 1 shows the schematic operation during memristor programming. The key elements that are involved in the write operation is highlighted in the figure, while other lines not used for writing are greyed out. From the schematic, one sees that the write operation is similar to that of a one-transistor one-memristor (1T1M) array: The DLs (data lines) select the memristor device to be programmed (as well as the aCAM array column), and the programming voltage is applied through the SL_hi and SL_lo row wires to set (program the device from a low conductance state to a high conductance state) or reset the the device (and select the aCAM array row). An analog voltage can be applied to DLs to set a compliance current during the set operation for a better multilevel tunability[1,2].

In the case that the programmed memristor conductance needs to be verified after the write operation, the conductance of a given memristor be read out by measuring the current while applying a reading voltage across SL_hi and SL_lo, with DL activated to select the device. SUPPLEMENTARY TABLE 1 summarizes the detailed voltage signals required for various operations.

Iterative programming of memristors may take a long time to complete and consume significant amount energy. However, the applications proposed in this work do not require frequent updates of memristor conductances and therefore the overhead of programming memristors is neg-

ligible. In addition, due to the nonvolatility of memristor devices, the analog CAM does not require frequent re-programming or stand-by power once programmed. In the case of applications that do require frequent updates (*e.g. in situ* training of a decision tree), the peripheral circuit design would need to be carefully designed and optimized.

Supplementary Note 2. Analysis on the effect of word length

The search operation of our analog CAM can be illustrated in SUPPLEMENTARY FIGURE 8. In the schematic, each dashed square represents a ML pull-down path in one analog CAM cell, which is a transistor whose channel conductance is low when the stored content matches the input and high when mismatches. Therefore, the ML stays high only when all the cells in a row match the given input, and the result is sensed by a sense amplifier attached to the ML. However, since all the pull-down paths are connected in parallel, when the word is long enough the overall pull-down conductance for a 'match' case could be higher than a mismatch worst case (i.e. a single-bit mismatch), leading to a sensing error and/or changes in the accepted search range of an analog CAM cell.

The problem is examined by quantitative analysis. The ML discharge process can be modelled by a RC decay or the discharging of a capacitor with a constant current sink (see SUPPLEMENTARY FIGURE 8). When all the analog CAM cells match the input, the equivalent conductance of the pull-down path is roughly $N \cdot G_{\text{T, OFF}}$, where N is the word length (i.e. width of the analog CAM array row). The pull-down path conductance for a mismatched case is larger or equal to $G_{\text{T, ON}} + (N-1) \cdot G_{\text{T, OFF}}$. A margin is required to differentiate the 'match' and the 'mismatch'

cases, which requires that the match case overall pull-down conductance is larger than that for a mismatch case. If we define the ratio of the conductance difference as β (>1), the requirement can be described in SUPPLEMENTARY EQUATION 1, and therefore the word length is limited by the conductance ON/OFF ratio (dynamic range) of the pull-down transistor.

$$G_{\mathrm{T,\,ON}} > [(\beta-1)N+1]\cdot G_{\mathrm{T,\,OFF}} \tag{1}$$

In addition, when the margin is large enough, the small pull-down leakage from the 'match' cells could change the search result as pointed out in the main text. This is because the conductance of the pull-down transistor is continuous with respect to the DL voltage ($G = f(V_{\mathrm{DL}})$), and so the search range for one analog CAM cell in an array is affected by other cells attached to the same ML, described in SUPPLEMENTARY EQUATION 2.

$$G_{\mathrm{T}} = f(V_{\mathrm{DL}}) < G_{\mathrm{th}} - (N-1)\cdot G_{\mathrm{T,\,OFF}} \tag{2}$$

where G_{th} is the criteria to differentiate the 'match' and 'mismatch' case. It is clear that other cells attached to the same ML equivalently shift the criteria, and therefore the search range. The amount of the change is reflected by $\partial G_{\mathrm{T}}/\partial V_{\mathrm{DL}}$, *i.e.* the conductance sensitivity to the change of analog voltage signal. The sensitivity can be written as $(\alpha S_s)^{-1}$, where α is the ratio between changes in V_{DL} and V_{G}, and S_s is the subthreshold swing slope of the transistor. As an example, considering a typical value for the V_{DL} to V_{G} ratio of 0.1 (simulated data shown in FIGURE 3d and 3e), and S_s of 100 mV/dec, the equation gives the overall sensitivity of about dec/10 mV. In simulation, the

change in the search range simulated in FIGURE 5 is tens of mV, which is fairly consistent with our analysis here. Therefore, employing the volatile threshold switching memristor with small sub-threshold swing great improves the performance, as simulated in FIGURE 9 and described in the main text.

Supplementary Note 3. Analog CAM with emerging threshold switching memristor

Typically, sub-threshold current leakage through the ML pull-down transistors limits the maximum CAM word length and the number of stored bits per cell. To improve the maximum CAM work length in our analog CAM, we propose to replace the standard ML pull-down transistor with volatile threshold switching (TS) memristors[3–5] which results in greatly reduced sub-threshold current leakage. Our analog CAM cell is converted to a circuit that is composed of four transistors, two non-volatile memristors, and two volatile TS memristors, as shown in SUPPLEMENTARY FIGURE 9a. In contrast to our first implementation using pull-down transistors to discharge the ML for a mismatch result, a search operation in this case starts with ML at ground, and the ML is charged up only for mismatch cases. The performance of the proposed cell is evaluated in simulation under 180 nm design rules which we used for the tapeout experiment. The TS memristor is modelled with Verilog-A on data extracted from published experimental data[3]. Although TS memristors in the literature may suffer from endurance limits, the following analysis is aimed at providing a direction for even further performance improvements, with future work needed to fully explore these trade-offs.

SUPPLEMENTARY FIGURE 9b shows the simulated current-voltage (IV) curve for the TS

memristor, from which one sees a significantly smaller sub-threshold swing than MOSFET transistors, thereby greatly decreasing the sub-threshold current leakage on the ML. The ML voltage sensed at 50 ns after the search starts (SUPPLEMENTARY FIGURE 9) shows a match for V_{DL} between 0.53 V and 0.65 V (see SUPPLEMENTARY SECTION 3 for additional details). As with the previous analog CAM cell, the simulated analog cell can be configured to match different V_{DL} ranges by programming different memristor conductances. SUPPLEMENTARY FIGURE 9d shows the successful analog CAM cell with 16 discrete programmable matching states. Further simulations (SUPPLEMENTARY FIGURE 9e and f) of the analog CAM arrays show that the change in the matching voltage range moves by less than 0.01 V with columns of up to 72, indicating the capability to store and search 5-6 bits of information, showing a significant improvement from conventional designs with pull-down transistors.

Supplementary Note 4. Comparison between the range search with TCAM and multibit analog CAM

Here we consider the CAMs that are used in a network router for classifying a random range in a 16-bit Class B IP address space (0-65535). In a TCAM, a continuous range can be represented by storing 'X' in the least significant bits - for example, 01XX is a range between the binary number of 0100 and 0111 (or decimal number 4-7). However, in most cases a random range will need to be split into multiple entries to fit in a TCAM. Taking a random range between 385 and 58630 (or hexadecimal number 0181 - E506), we observe that this range can be implemented by a 21×16 TCAM array as shown in SUPPLEMENTARY FIGURE 11. Although it may seem to be an acceptable overhead in this example here, this may not be the case when the ranges in a larger

space are considered (e.g. 128 bit for IPv6). Here, we restrict ourselves to this smaller example for comparison purposes.

First, we observe that the whole TCAM array described above for a range search can be replaced by a single 16-bit analog cell. With the present design, it is challenging to realize a 16-bit analog CAM cell currently, but we've shown that it is very feasible for a 3-bit or 4-bit cell to be implemented, as demonstrated in FIGURE 3e,f. With limited bit-precision analog CAM cells, the range can be split in a similar way as in TCAM entries. SUPPLEMENTARY FIGURE 11b and SUPPLEMENTARY FIGURE 11c show implementations with 3-bit analog CAM and 4-bit analog CAM respectively. In the table, 'X' is similar to that in the TCAM, which matches everything, i.e. 0-7 in a 3-bit cell or 0-15 in a 4-bit cell. $\{n-m\}$ represent the cell is configured to match part of the range between n and m. From the figure, one sees that by using a multi-bit analog CAM, both columns and rows can be compressed, leading to a reduction from 336 TCAM cells to 54 3-bit cells, 24 4-bit cells, or 6 8-bit cells. In addition, there are only six transistors in an analog CAM cell, while 16 in a SRAM-based one. With these factors taken into consideration, the overall transistor count required for this specific function results in a 37× reduction. Assuming a TCAM implementation with $0.70\,\mu m^2$ per TCAM cell area overhead in a standard library under the same 16 nm technology node, the proposed range search functionality occupies $235.20\,\mu m^2$ chip area while only $12.48\,\mu m^2$ with our analog CAM, leading to 18.8× reduction in area. The decreased area reduction comparing to that in transistor count is due to the fact that the reference SRAM layout utilizes the foundry's SRAM-specific layout rules, while our present analog CAM layout follows a more conservative logic rule. We also expect a similar reduction in operational

power with the analog CAM cell in comparison to conventional TCAMs, as a major portion of the dynamic energy consumption for a CAM operation is charging parasitic wire capacitances, and the reduced cell count and area also results in shorter wires and reduces total wire capacitance.

Supplementary Note 5. Energy estimation with a custom designed digital-to-analog converter

To evaluate the search energy consumption, our analog CAM array simulations measure the consumed power during search operations by integrating current from all power supplies. As the evaluated IP routing application handles digital signals, digital-to-analog converters (DAC) are included in the analysis. The DACs impose additional overhead in terms of both chip area and energy, but the analysis below shows that the DAC overhead is not overwhelming. Importantly, for applications handling analog signals directly, digital-analog signal conversion is not required at all with our analog CAM, suggesting a promising application space. In contrast, analog-to-digital (ADC) converters would be required when using digital SRAM-based TCAMs, and we note ADCs are usually much more expensive (area/power) than DACs.

The DAC we use in our evaluation is a simple 4-bit current-steering design, the schematic of which is shown in SUPPLEMENTARY FIGURE 12. There are two major portions in the DAC design, with the first being the circuit mirrors input for $I_{\mathrm{REF}} \times 1$ and $I_{\mathrm{REF}} \times 4$ respectively (shown in panel a). The input current mirrors are shared globally across all the DAC channels, while the current mirror outputs are selectively turned on to convert the digital inputs that are applied on the switch transistors to analog outputs (panel b). The simulated curves for the DAC operation are shown in SUPPLEMENTARY FIGURE 12c.

While unoptimized, the total energy consumption during search operations in an 86×12 analog CAM array is estimated to be 0.52 fJ per search per cell according to the simulations (SUPPLEMENTARY TABLE 2), and will be smaller in practice due to larger arrays and their reduced average DAC peripheral cost. If we convert the performance number in the aforementioned search table for IP routing, the same search function in the 21×16 TCAM table will consume 12.48 fJ per search in a 6×4 analog array, leading to a 0.037 fJ per search per equivalent TCAM bit. Additional energy improvements are expected for applications that handle analog signals directly.

Supplementary Note 6. Decision Tree model mapped in our proposed analog CAM

Decision trees with binary and non-binary classification features can be implemented in the analog CAM directly by mapping each root to leaf path to a row in the analog CAM. Logically, each root-to-leaf path traverses a series of nodes with Boolean ANDs between elements in a given input feature vector (SUPPLEMENTARY FIGURE 13a). Since AND is commutative, we can reorder the nodes such that feature variables are processed in the same order for all paths[6]. Nodes for the same feature are combined into one node and "don't care" nodes can be inserted for features absent from a specific path, such that each path is of equal length. This representation can then be directly mapped to the analog CAM array, with each root to leaf path a row (see SUPPLEMENTARY FIGURE 13). As the matching row can directly drive the readout of the classification result, tree traversal becomes a one-cycle operation (SUPPLEMENTARY FIGURE 13b). As each of the split outcomes of the tree are mutually exclusive, only one root-to-leaf path, or one row in the analog CAM, will "match" for a given feature vector. A collection of analog CAM arrays for decision trees with a local direct classification lookup can be used to implement ensemble tree-based models which are

popular machine learning models.

4 Supplementary References

-

1. Li, C. *et al.* Analogue signal and image processing with large memristor crossbars. *Nature Electronics* **1**, 52 (2018).

2. Hu, M. *et al.* Memristor-based analog computation and neural network classification with a dot product engine. *Advanced Materials* (2018).

3. Midya, R. *et al.* Anatomy of ag/hafnia-based selectors with 10^{10} nonlinearity. *Advanced Materials* **29**, 1604457 (2017).

4. Kim, S. *et al.* Ultrathin ($<$ 10nm) Nb_2O_5/NbO_2 hybrid memory with both memory and selector characteristics for high density 3d vertically stackable rram applications. In *2012 Symposium on VLSI Technology (VLSIT)*, 155–156 (IEEE, 2012).

5. Son, M. *et al.* Excellent selector characteristics of nanoscale VO_2 for high-density bipolar reram applications. *IEEE Electron Device Letters* **32**, 1579–1581 (2011).

6. Buschjäger, S. & Morik, K. Decision tree and random forest implementations for fast filtering of sensor data. *IEEE Transactions on Circuits and Systems I: Regular Papers* **65**, 209–222 (2017).
